# Supplementary material for: Pyruvate Dehydrogenase Kinase 1 inhibition mediated oxidative phosphorylation enhancement in cartilage promotes osteoarthritis progression
Source: BMC Musculoskelet Disord. 2023 Jul 20;24:597. doi: 10.1186/s12891-023-06585-6 (PMC10357736; doi:10.1186/s12891-023-06585-6)
Supplement: Supplementary file 1 — Additional file 1. [file 12891_2023_6585_MOESM1_ESM.zip › BP.Gsea.1653623667859/GOBP_CELL_PROLIFERATION_INVOLVED_IN_KIDNEY_DEVELOPMENT.html]

Details for gene set GOBP\_CELL\_PROLIFERATION\_INVOLVED\_IN\_KIDNEY\_DEVELOPMENT[GSEA]

|  || Dataset | 55457\_collapsed\_to\_symbols.cls.cls #OA\_versus\_normal.cls.cls #OA\_versus\_normal\_repos |
| Phenotype | cls.cls#OA\_versus\_normal\_repos |
| Upregulated in class | normal |
| GeneSet | GOBP\_CELL\_PROLIFERATION\_INVOLVED\_IN\_KIDNEY\_DEVELOPMENT |
| Enrichment Score (ES) | -0.5723074 |
| Normalized Enrichment Score (NES) | -1.7606838 |
| Nominal p-value | 0.0070921984 |
| FDR q-value | 0.11051466 |
| FWER p-Value | 0.998 |
Table: GSEA Results Summary

  

Fig 1: Enrichment plot: GOBP\_CELL\_PROLIFERATION\_INVOLVED\_IN\_KIDNEY\_DEVELOPMENT      
 Profile of the Running ES Score & Positions of GeneSet Members on the Rank Ordered List

  

| SYMBOL | TITLE | RANK IN GENE LIST | RANK METRIC SCORE | RUNNING ES | CORE ENRICHMENT || 1 | LIN28A | lin-28 homolog A [Source:HGNC Symbol;Acc:HGNC:15986] | 3140 | 0.236 | -0.2103 | No |
| 2 | PDGFD | platelet derived growth factor D [Source:HGNC Symbol;Acc:HGNC:30620] | 3850 | 0.171 | -0.2412 | No |
| 3 | STAT1 | signal transducer and activator of transcription 1 [Source:HGNC Symbol;Acc:HGNC:11362] | 4495 | 0.113 | -0.2752 | No |
| 4 | WT1 | WT1 transcription factor [Source:HGNC Symbol;Acc:HGNC:12796] | 5452 | 0.037 | -0.3442 | No |
| 5 | PDGFRB | platelet derived growth factor receptor beta [Source:HGNC Symbol;Acc:HGNC:8804] | 6914 | -0.073 | -0.4473 | No |
| 6 | GPC3 | glypican 3 [Source:HGNC Symbol;Acc:HGNC:4451] | 7097 | -0.087 | -0.4490 | No |
| 7 | SERPINB7 | serpin family B member 7 [Source:HGNC Symbol;Acc:HGNC:13902] | 7679 | -0.133 | -0.4753 | No |
| 8 | BMP4 | bone morphogenetic protein 4 [Source:HGNC Symbol;Acc:HGNC:1071] | 7816 | -0.142 | -0.4657 | No |
| 9 | PTCH1 | patched 1 [Source:HGNC Symbol;Acc:HGNC:9585] | 8837 | -0.220 | -0.5137 | Yes |
| 10 | FLCN | folliculin [Source:HGNC Symbol;Acc:HGNC:27310] | 8848 | -0.221 | -0.4832 | Yes |
| 11 | SHH | sonic hedgehog signaling molecule [Source:HGNC Symbol;Acc:HGNC:10848] | 8941 | -0.227 | -0.4582 | Yes |
| 12 | BMP7 | bone morphogenetic protein 7 [Source:HGNC Symbol;Acc:HGNC:1074] | 9544 | -0.278 | -0.4656 | Yes |
| 13 | BMP2 | bone morphogenetic protein 2 [Source:HGNC Symbol;Acc:HGNC:1069] | 10919 | -0.420 | -0.5128 | Yes |
| 14 | IL6R | interleukin 6 receptor [Source:HGNC Symbol;Acc:HGNC:6019] | 11615 | -0.519 | -0.4933 | Yes |
| 15 | EGR1 | early growth response 1 [Source:HGNC Symbol;Acc:HGNC:3238] | 12052 | -0.607 | -0.4412 | Yes |
| 16 | CFLAR | CASP8 and FADD like apoptosis regulator [Source:HGNC Symbol;Acc:HGNC:1876] | 12079 | -0.614 | -0.3563 | Yes |
| 17 | PDGFA | platelet derived growth factor subunit A [Source:HGNC Symbol;Acc:HGNC:8799] | 12104 | -0.621 | -0.2702 | Yes |
| 18 | GATA3 | GATA binding protein 3 [Source:HGNC Symbol;Acc:HGNC:4172] | 12276 | -0.670 | -0.1887 | Yes |
| 19 | PDGFB | platelet derived growth factor subunit B [Source:HGNC Symbol;Acc:HGNC:8800] | 12389 | -0.704 | -0.0977 | Yes |
| 20 | MYC | "MYC proto-oncogene, bHLH transcription factor [Source:HGNC Symbol;Acc:HGNC:7553]" | 12801 | -0.970 | 0.0077 | Yes |
Table: GSEA details [plain text format]

  

Fig 2: GOBP\_CELL\_PROLIFERATION\_INVOLVED\_IN\_KIDNEY\_DEVELOPMENT      
 Blue-Pink O' Gram in the Space of the Analyzed GeneSet

  

Fig 3: GOBP\_CELL\_PROLIFERATION\_INVOLVED\_IN\_KIDNEY\_DEVELOPMENT: Random ES distribution      
 Gene set null distribution of ES for **GOBP\_CELL\_PROLIFERATION\_INVOLVED\_IN\_KIDNEY\_DEVELOPMENT**
